# Supplementary material for: Splice-Junction-Based Mapping of Alternative Isoforms in the Human Proteome
Source: Cell Rep. Author manuscript; Available in PMC 2020 Jan 15. (PMC6961840; doi:10.1016/j.celrep.2019.11.026)
Supplement: 2 [file NIHMS1546469-supplement-2.zip › DF1/PXD006675/MitralValve/MitralValve_8_CYBA_KPSEEEAAVAAGGPPGGPQVNPIPVTDEVDR.pdf]

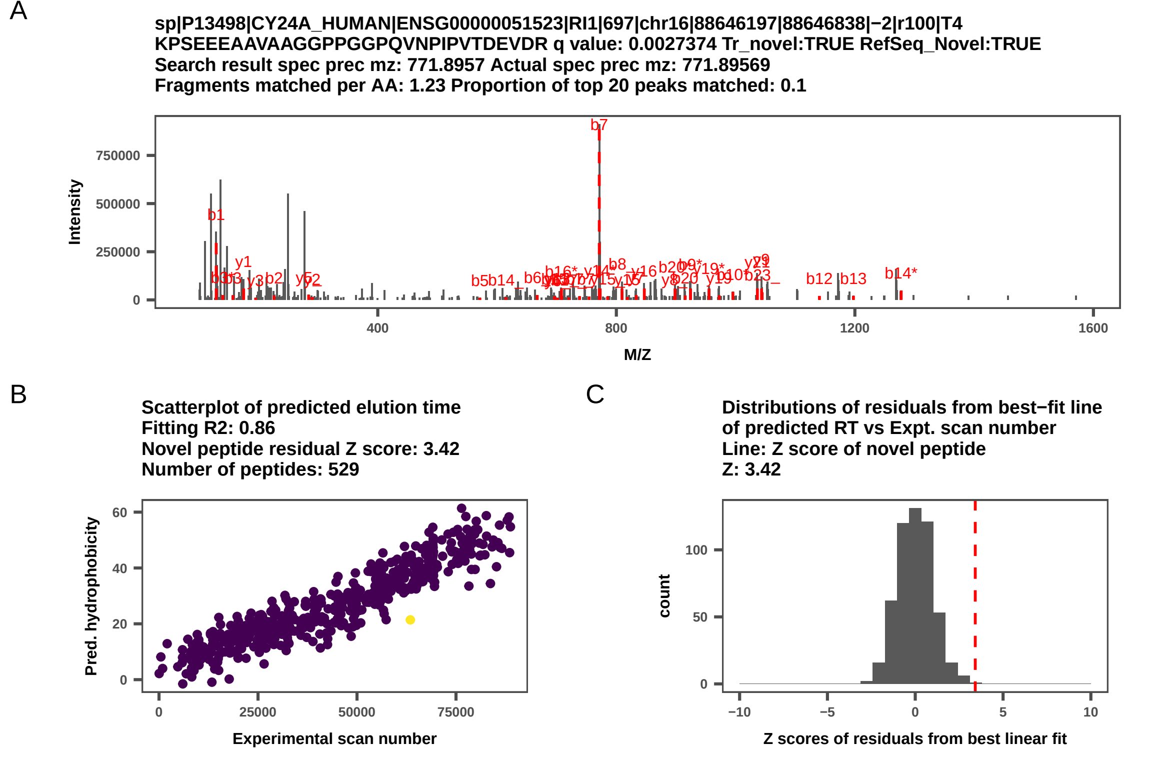
A
sp|P13498|CY24A_HUMAN|ENSG00000051523|RI1|697|chr16|88646197|88646838|−2|r100|T4
KPSEEEAAVAAGGPPGGPQVNPIPVTDEVDR q value: 0.0027374 Tr_novel:TRUE RefSeq_Novel:TRUE
Search result spec prec mz: 771.8957 Actual spec prec mz: 771.89569
Fragments matched per AA: 1.23 Proportion of top 20 peaks matched: 0.1
Intensity
750000
500000
250000
0
400
800
1200
1600
M/Z
b7
b1
y1
b2
b8
b12
b13
b14*
B
Scatterplot of predicted elution time
Fitting R2: 0.86
Novel peptide residual Z score: 3.42
Number of peptides: 529
Pred. hydrophobicity
60
40
20
0
25000
50000
75000
Experimental scan number
C
Distributions of residuals from best−fit line
of predicted RT vs Expt. scan number
Line: Z score of novel peptide
Z: 3.42
count
100
50
−10
−5
5
10
Z scores of residuals from best linear fit
